# Relationship between manufacturing complexity, strategy, and performance of manufacturing industries in Indonesia

**DOI:** 10.1016/j.heliyon.2021.e07225

**Published:** 2021-06-05

**Authors:** Hendri Dwi Saptioratri Budiono, Rahmat Nurcahyo, Muhammad Habiburrahman

**Affiliations:** a Department of Mechanical Engineering, Faculty of Engineering, Universitas Indonesia, Kampus Baru UI Depok, 16424 Indonesia; b Department of Industrial Engineering, Faculty of Engineering, Universitas Indonesia, Kampus Baru UI Depok, 16424 Indonesia

**Keywords:** Manufacturing complexity, Manufacturing performance, Manufacturing strategy, Quality strategy

## Abstract

Previous research has emphasized the need to further investigate the impact of manufacturing complexity on company strategies and performance in developing countries' manufacturing sector. Indonesia is one such developing country where this relationship has yet to be adequately studied. The manufacturing industry sector is expected to drive Indonesia's economic growth to achieve the targeted average growth of 6% per year in the next five years. This study aimed to examine the relationship between manufacturing complexity, manufacturing strategies (cost, delivery, flexibility, and quality), and manufacturing performance of Indonesian automotive component manufacturing industries. Partial least square structural equation modeling (PLS-SEM) was employed in the investigation. The sample size comprised 72 automotive component manufacturing companies located in the Jakarta region. Results indicated that the higher the value of manufacturing complexity, the higher the manufacturing strategies should be prioritized. The manufacturing strategy related to quality, particularly, has a significant positive impact on manufacturing performance. The novelty of this study lies in its detailed examination of the relationship between manufacturing complexity, manufacturing strategies, and manufacturing performance, particularly in developing countries. The results are expected to fill the existing research gaps.

## 1 Introduction

### 1.1 Indonesia's manufacturing industry

According to Indonesia Law No. 3 of 2014 on Industry, one of Indonesia's industrialization goals is for the nation to act as a pillar and driver of the national economy. Industrial development is directed at realizing a competitive industry that is maintained in an integrated and sustainable manner to provide benefits to society (Ministry of Industry, 2015).

The manufacturing industry is an industrial sector that provides the largest contribution to the Indonesian economy (Ministry of Industry, 2020a). The manufacturing industry sector is expected to drive Indonesia's economic growth to achieve the targeted average growth of 6% per year in the next five years (Asian Development Bank and Bappenas, 2019). According to the data from the Indonesia Central Statistics Agency (*Badan Pusat Statistik* or BPS), the production of the manufacturing industry in 2012–2019 grew but at a slower rate than that during its peak in 2013 (Central Statistics Agency, 2020). In 2015–2019, the manufacturing industry's average growth in terms of production reached 4.3%, which is below the average national economic growth rate (5% on average) (Central Statistics Agency, 2020). The role of the manufacturing industry in BPS records is still the largest, with its share making up 19.86% of the GDP (Ministry of Industry, 2020a). In 2020, the targeted manufacturing industry growth was 5.3%, which should equate to a 17.8% contribution to the total national GDP (Ministry of Industry, 2020b). Indonesia needs to attain higher growth rates. Therefore, the manufacturing sector has to play a more important role in driving Indonesia's structural transformation (Asian Development Bank and Bappenas, 2019).

The COVID-19 pandemic continues to exert a significant impact on the Indonesian economy. In 2020, Indonesia entered a recession after the national economy contracted for two consecutive quarters, namely, minus 5.32% during the second quarter and minus 3.49% in the third quarter of 2020 (World Bank, 2020). The impact on the manufacturing industry in Indonesia varies between industrial sectors. *Kementerian Perindustrian* or Ministry of Industry in their press release stated that the contraction in the manufacturing sector was mainly influenced by the decline in domestic demand which has beenable to absorb up to 70% of the total production of the domestic manufacturing industry (Ministry of Industry, 2020c).

According to an article published by the Indonesian Ministry of Industry through its official website, the Indonesian government is known to be optimistic about the growth of the country's manufacturing industry and its development that can make Indonesia a hub or manufacturing center in ASEAN (Ministry of Industry, 2019). This description rings true because Indonesia is one of the leading destination countries for various manufacturing companies seeking to make investments and fulfill import and export demands (Fernandez et al., 2020). However, Indonesia's policy makers acknowledge today that it would be very difficult to realize it without achieving substantially higher levels of industrialization (Asian Development Bank and Bappenas, 2019). Yet they find it very difficult to diversify and upgrade the manufacturing industry to make it the engine of growth (Asian Development Bank and Bappenas, 2019).

The manufacturing industry cannot be separated from market uncertainty, technological innovation trends, dynamic environmental conditions, market globalization, competition, and changing customer requirements. As the industry develops, challenges in the manufacturing environment also increase. One of the main challenges is manufacturing complexity (Mahmood et al., 2015). Identifying, analyzing, and understanding the drivers of manufacturing complexity are the first steps toward developing and implementing complexity management (Vogel and Lasch, 2016). Manufacturing complexity management is a strategic issue that companies need to tackle to participate in a competitive environment (Vogel and Lasch, 2016).

The driving factors for complexity in the manufacturing system are divided into external and internal factors (Vogel and Lasch, 2016). External factors consist of the supply network and market aspects. The supply network aspect includes large numbers of organizations, complex interactions, and decentralized networks. The market aspect includes large demand, commodity fluctuations, and buyer behavior. The internal factors consist of system and product aspects. The system aspect includes increased flexibility, complicated configurations, and machine breakdowns. The product aspect comprises increased product models and choices and increased number of product constituent parts (Efthymiou et al., 2016).

The current study examines manufacturing complexity, especially that in the automotive component industry in Indonesia which is one of the drivers of Indonesia's manufacturing industry (Asian Development Bank and Bappenas, 2019). The automotive industry is an important industry because of the numerous derivatives in its supply chain and the participation and support of small and medium industries. Further development and exploitation of the automotive industry may be an appropriate development strategy (Asian Development Bank and Bappenas, 2019). Also, product complexity is relatively high in a number of sectors, for example, automotive and machinery and equipment (Asian Development Bank and Bappenas, 2019).

With the aforementioned background and challenges, this research question is arises: What is the relationship between manufacturing complexity, strategy and performance? To answer the research question, this study aimed to examine the relationship between manufacturing complexity, manufacturing strategies (cost, delivery, flexibility, and quality), and manufacturing performance of Indonesian automotive component manufacturing industries.

## 2 Literature review

### 2.1 Manufacturing system complexity

To meet production targets with increasingly complex products, high quality requirements, and short marketing times, the manufacturing industry uses highly advanced production systems and various subsystems. These advances and modifications have increased manufacturing systems’ complexity down to the factory floor (Alkan et al., 2018). According to ElMaraghy and Urbanic (2003), the complexity of a manufacturing system increases not only with the increasing number and variety of features to be produced, assembled, and tested but also with the many types and processes involved to produce these features. That is, complexity increases as the number of production facilities in a manufacturing system increases. Furthermore, complexity and the occurrence of failures in manufacturing systems are closely related (ElMaraghy et al., 2012).

The research on manufacturing complexity enables the design and planning of other productive and predictive manufacturing systems (Efthymiou et al., 2016). The analysis of manufacturing systems’ complexity leads to an increase in reliable manufacturing systems (Garbie and Shikdar, 2010).

Physical and functional complexity are two domains of complexity in manufacturing systems (ElMaraghy et al., 2012). In the physical domain, complexity is divided into two categorizes: static and dynamic (Frizelle and Woodcock, 1995; Efthymiou et al., 2012). Static, also called structural complexity, reflects a time-independent characteristic of manufacturing systems and focuses on the type of subsystem and interconnection strength (Deshmukh et al., 1998). Static complexity is related to systems’ structure and configuration; the number and variety of products; and the variety, interconnection, and interdependence of system components, such as labor, machines, support, and transportation mechanisms (Efthymiou et al., 2012). Dynamic or operational complexity is a characteristic of system operations and involves aspects of time and randomness (Frizelle and Suhov, 2008). Dynamic complexity is related to the uncertainty of system behavior for a certain period and to the possibility of the system being controlled (Efthymiou et al., 2012).

An increase in manufacturing system complexity has been stated to have a negative impact on all areas of manufacturing, including product quality, reliability, throughput, and lead time, as well as disrupting system productivity at the design, production, maintenance, and management levels (Schuh et al., 2015). Complexity also negatively impacts the company's indicators and generates losses related to profits, revenues, sales volume and customer losses (Bozarth et al., 2009). Furthermore, within manufacturing processes, uncertainty and the incidence of failure are inextricably linked (ElMaraghy et al., 2012). In manufacturing systems, it is important to mitigate complexity (Vidal and Hernandez, 2021), an increase in manufacturing system complexity is only reasonable if it boosts the system's capabilities, features, usability, and performance (Samy et al., 2015). To stay profitable and competitive, and to adapt quickly to dynamic markets and increasing product diversity, uncertainty and its effect on system key performance indicators (KPIs) should be identified and quantified (Mattsson et al., 2011). In order to do so, a thorough study and evaluation of complexity, as well as identification of its consequences, is needed (Alkan et al., 2018). As a result, key managerial aspects are highlighted, allowing for the implementation of strategies to manage system complexity.

The factors causing complexity may originate from external and internal (Isik, 2010). External complexity depends on variables between downstream associated with customer and upstream with respect to supplier while internal complexity relates to variables between flows within manufacturing (Bozarth et al., 2009). Modern manufacturing systems operate in an ambiguous and constantly evolving environment, influenced by global, socioeconomic, and political influences (ElMaraghy et al., 2013). External complexity, such as demand uncertainty and volatility, advancements in technology, global rivalry, and supplier variability, significantly impact them (Alkan et al., 2018). These drivers could be connected to a company's internal complexity, where considerations like a large number of heterogeneous customers, large product portfolios, increased product complexity, and a large number and variety of market priorities are mainly leverage the internal complexity (Marti, 2007). As a result, there is more complexity in manufacturing systems, which leads to more data generation and unpredicted/unknown behaviors (ElMaraghy et al., 2013).

Product variety needs a higher degree of flexibility for handling parts due to changes in technical and functional features of the products, such as configuration, size and shape (Chinnathai et al., 2017). Dealing with demand uncertainty forces the system to respond and adapt, resulting in challenges with stochastic line balance (Bilge et al., 2015). On the other side, when cycle times become more important in the production system, increased demand needs more complicated machine design and more machines, returning producers to the line balance issue (Fathi et al., 2016). To assess process quality, high-quality requirements involve more quality-control systems inside the production system, which may expand the number of stations or even the complexity of a single station. Moreover, quality data management, analysis, and suitable exploitation all add to the complexity of the production system.

### 2.2 Manufacturing strategy

Various studies have explored the formulation of manufacturing strategies with a focus on design and planning (Kim and Arnold, 1996). Various dimensions of manufacturing strategies have also been developed (Spring and Dalrymple, 2000), and they are consistent with the following four variables first described by Skinner: cost, quality, delivery, and flexibility (In Wheelwright, 1978). These four factors are at the core of any manufacturing strategy. The reason for choosing these four variables is because cost, quality, delivery and flexibility are the dimensions of manufacturing strategy that are most commonly found to have a strong and positive relationship with company success based on research that has been done (Terjesen et al., 2010).

The first is cost strategy. Manufacturing companies tend to achieve economies of scale and benefit from cheaper buying, distribution, and selling costs. Costs are not defined as mere low costs but all the taken means to reduce, even if possible, eliminate waste through a cost reduction program. The cost dimension in manufacturing strategy is related to production and distribution costs (Dangayach and Deshmukh, 2001) and can be measured by reducing production costs, reducing inventory, increasing machine utilization and capacity (Ward and Duray, 2000). Rose et al. (2008) describe this dimension as a cost-based strategy. One focus on a cost-based strategy is labor because of the factors that affect the production process but are the most costly and relatively difficult to control. What can be done is to utilize technology to reduce dependence on humans. Another focus in this strategy is reducing inventory, leading to just-in-time (JIT) implementation and reducing production time, leading to process improvements.

The second is delivery strategy. Delivery is a service to customers, generally indicated by the speed and reliability of the delivery (Ward and Duray, 2000). Delivery is considered a time-based problem (Phusavat and Kanchana, 2007). The essence is to take advantage of speed to gain a competitive advantage by delivering products and services faster than competitors. Delivery involves the ways to quickly provide products or services to customers (Li, 2000). Shipping also incorporates a time-to-market point of view for new products. Reeves and Bednar (1994) attributed quality to excellence, value, conformity to specifications, and capability of meeting or exceeding customer expectations. The application of speed in a manufacturing strategy is known as quick response manufacturing or QRM. Externally, this means providing a quick response to customer needs through the speed of design and manufacturing. Internally, this will focus on reducing the lead time in every activity of the organization.

The third is flexibility strategy. Flexibility is defined as a manufacturing system's ability to respond effectively and quickly to changing production needs and requirements (Kaschel and Bernal, 2006). Manufacturing companies have to address the challenge posed by variable demand (Kusiak, 2019). Several manufacturers have sought to improve the flexibility of their manufacturing facilities so that production volume and variety can be more readily adapted to meet changing demand (Phan et al., 2019). This capability is becoming increasingly important for manufacturing systems' design and operation as these systems operate in highly variable and unpredictable environments. The combined impact of three pairs of TQM and JIT manufacturing practices: process control and setup time reduction, supplier participation and JIT delivery by suppliers, and customer involvement and JIT link with customers, according to a recent study by Phan et al. (2019).

The Fourth is quality strategy. The quality strategy is defined as any means to provide customers with added value, which should exceed consumer expectations as much as possible (Lowson, 2002). Quality is multidimensional, and each dimension can be used strategically for competitive advantage (Garvin, 1987). This strategy is one of the main competitive priorities for automotive component companies (Laosirihongthong and Dangayach, 2005). The main objective of organizations adopting a strategic management process is to explore the factors that affect performance improvement (Man, 2009). Cooperation of all employees is a critical element (Rose et al., 2008) because the implementation of quality factors in manufacturing requires total commitment and involvement of top management and all employees. Significantly, quality will improve product and process design, implementation of process control, and continuous improvement of work processes (Rose et al., 2008).

Automotive component companies implement the four dimensions of the manufacturing strategy through various production system techniques and quality systems. This research uses the paradigm of manufacturing strategy trade-off, which means that automotive component companies will only focus on one manufacturing strategy dimension.

Manufacturing strategies play an important role in manufacturing industry systems’ complexity through concepts such as just-in-time production and lean manufacturing. System complexity for lean manufacturing, for example, is influenced by an increase in product variation relative to the mass production system (Garbie and Shikdar, 2010). Analyzing and understanding manufacturing complexity facilitates the development and implementation of appropriate strategies for complexity management (Efthymiou et al., 2016).

## 3 Methods

### 3.1 Model development

In studying the relationship between complexity, strategy, and performance, the dependent and independent variables should be identified. Complexity is defined as an independent variable, while performance is classified as a dependent variable. The cost, delivery, flexibility, and quality constructs are endogenous and have dual relationships as both independent and dependent. They are dependent constructs because they are predicted by complexity, but they are also independent constructs because they predict performance. The path relations of the variables are shown in Tables 1 and 2.

**Table 1:** Path relations: complexity to strategy.

Path	Reference
Complexity - Cost	Ma et al. (2012); Schuh et al. (2015); Asl and Ulsoy (2003); Danese and Kalchschmidt (2011); Hwarng and Xie (2008); Alkan et al. (2018); Efthymiou et al. (2012).
Complexity - Flexibility	Ma et al. (2012); Garbie and Shikdar (2010); Schuh et al. (2015); Mattsson et al. (2011); Alkan et al. (2018); Efthymiou et al. (2012).
Complexity - Delivery	Ma et al. (2012); Alkan et al. (2018); Schuh et al. (2015); Mattsson et al. (2011); Efthymiou et al. (2012).
Complexity - Quality	Ma et al. (2012); Alkan et al. (2018); Schuh et al. (2015).

**Table 2:** Path relations: strategy to performance.

Path	Reference
Cost - Performance	Baum et al. (2000); Badri et al. (2000); Covin et al. in Terjesen et al. (2010).
Flexibility - Performance	Machuca et al. (2011); Swamidass and Newell (1987); White (1996).
Delivery - Performance	Laosirihongthong and Dangayach (2005); Rose et al. (2008); Wood et al. (1990).
Quality - Performance	Amoako-Gyampah and Acquaah (2008); Ferdows and De Meyer (1990); Terjesen et al. (2010).

**Figure 1:**
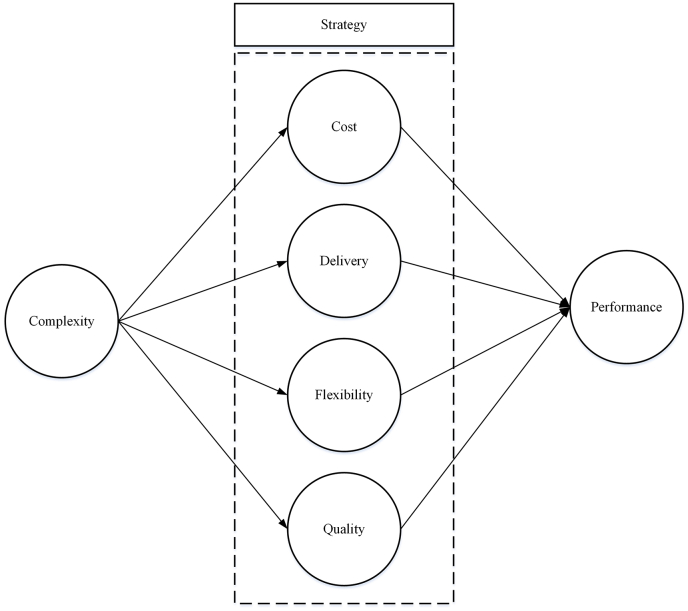
Research conceptual model.

According to the model depicted in Figure 1, the research hypotheses can be constructed as follows:

H1: The higher the value of manufacturing complexity, the higher the value of cost strategy should be prioritized.
H2: The higher the value of manufacturing complexity, the higher the value of delivery strategy should be prioritized.
H3: The higher the value of manufacturing complexity, the higher the value of flexibility strategy should be prioritized.
H4: The higher the value of manufacturing complexity, the higher the value of quality strategy should be prioritized.
H5: The manufacturing cost strategy has a positive impact on performance.
H6: The manufacturing delivery strategy has a positive impact on performance.
H7: The manufacturing flexibility strategy has a positive impact on performance.
H8: The manufacturing quality strategy has a positive impact on performance.

### 3.2 Data collection and processing

After the construction of the research's conceptual model (Figure 1), the questionnaire for data collection was then created (Appendix 1). The questionnaire consists of four sections. Section 1 involves general data, such as personal and company information. Section 2 deals with company complexity assessment(s). Section 3 accommodates the questions on company manufacture strategies related to cost, flexibility, delivery, and quality. Section 4 contains questions related to company performance. The number of scale points to be used was not set; most studies use four to seven points. Only six-point scales follow normal distributions (Leung, 2011). Therefore, each question in the questionnaire uses a six-point Likert scale.

Cohen produced a table for researchers to determine the sample size of their research using partial least square (PLS) structural equation modeling (SEM) (In Hair et al., 2016). In this model, there are four arrows pointing at a construct, therefore, this research required 65 samples to reach an 80 percent statistical power for identifying R^2^ values of at least 0.25 with a 0.05 chance of error (Cohen in Hair et al., 2016). The questionnaire was distributed to automotive component manufacturing companies in the Jakarta Greater Area. A total of 72 responses were recorded (Table 3).

**Table 3:** Descriptive statistics of respondents.

		Respondent	Percentage
Company age	<5 years	7	9.7
	5–10 years	6	8.3
	>10 years	59	81.9
Number of workers	<100 Workers	6	8.3
	100 - 500 Workers	10	13.8
	>500 Workers	56	77.7
Respondent age	<30 years	39	54.1
	30–40 years	29	40.2
	>40 years	4	5.5
Respondent position	Staff	29	40.2
	Supervisor	31	43
	Associate Manager	4	5.5
	Manager	8	11.1
Respondent experience	<5 years	26	36.1
	5–10 years	33	45.8
	>10 years	13	18

The data were then processed via PLS-SEM. In research, SEM is a powerful statistical approach for identifying the relationships between variables (Hair et al., 2016). PLS-SEM is a variance-based structural equation model estimation approach. The objective is to increase the amount of endogenous latent variables that can be described (Hair et al., 2016). PLS offers excellent capabilities for work with small samples as the methodology is sufficient for most success factor (cause indicator) analyses (Henseler et al., 2009). PLS-SEM could be a very useful analysis tool for future theory development especially based on its suitability for exploratory research questions (Nitzl, 2016). Therefore, PLS-SEM is the suitable method for this study.

### 3.3 Model testing

Composite reliability, individual indicator reliability, and average variance extracted (AVE) are all used to evaluate the measurement model's internal consistency and convergent validity. Additionally, the Fornell–Larcker criterion and cross-loadings are used to assess discriminant validity.

The first data processing resulted in the deletion of COMP4 “Uncertainty in increasing product variety,” COMP6 “There are deviations in the use of resources scheduled with the realization,” and COMP7 “Difficulty in determining key performance indicators (KPI)” because the outer loading values were lower than 0.40 and the complexity's AVE was below 0.50. Table 4 shows the results of the model's refinement.

**Table 4:** Result summary of models.

Latent Variable	Indicators	Loadings	Indicator Reliability	Composite Reliability	AVE	Discriminant Validity?
Complexity	COMP1	0.866	0.749	0.924	0.754	Yes
	COMP2	0.904	0.817			
	COMP3	0.890	0.792			
	COMP5	0.810	0.656			
Cost	COST1	0.887	0.786	0.925	0.754	Yes
	COST2	0.846	0.715			
	COST3	0.880	0.774			
	COST4	0.860	0.740			
Delivery	DELI1	0.848	0.719	0.879	0.784	Yes
	DELI2	0.921	0.848			
Flexibility	FLEX1	0.861	0.741	0.932	0.734	Yes
	FLEX2	0.856	0.733			
	FLEX3	0.889	0.790			
	FLEX4	0.883	0.780			
	FLEX5	0.790	0.624			
Quality	QUAL1	0.928	0.861	0.958	0.693	Yes
	QUAL2	0.876	0.767			
	QUAL3	0.880	0.774			
	QUAL4	0.918	0.843			
	QUAL5	0.925	0.856			
Performance	PERF1	0.806	0.650	0.947	0.82	Yes
	PERF2	0.819	0.671			
	PERF3	0.832	0.692			
	PERF4	0.832	0.692			
	PERF5	0.915	0.837			
	PERF6	0.827	0.684			
	PERF7	0.771	0.594			
	PERF8	0.852	0.726			

According to a common rule of thumb for indicator reliability, a latent variable should explain a significant part, usually at least 50%, of each indicator's variance (Hair et al., 2016). Therefore, the outer loading of an indicator should be more than 0.708, because that value squared (0.7082) equals 0.50. All the indicators for the six constructs in this work were well above the minimum acceptable level for outer loadings (Table 4). The composite reliability varied from 0 to 1, with greater values indicating greater levels of reliability. Specifically, the composite reliability values of 0.60–0.90 can be regarded as satisfactory (Nunally and Bernstein in Hair et al., 2016). Hence, all six constructs in this work were satisfactory in terms of composite reliability (Table 4).

According to empirical standards, discriminant validity refers to how different a construct is from other constructs. As a result, discriminant validity denotes that a construct is distinct and captures phenomena that are not represented by other constructs in the model. One method for assessing discriminant validity is by examining the cross-loadings of the indicators. The outer loading of an indicator on the related construct should be higher than its loadings on all other constructs. Herein, all six constructs satisfied the discriminant validity (Table 4).

Therefore, all the indicators and constructs were reliable and valid.

## 4 Results

### 4.1 Assessment of structural model

The structural model is evaluated by examining at its predictive capabilities as well as the relationships between the constructs. The significance of the path coefficients, level of R^2^ values, F^2^ effect size, predictive relevance, and Q^2^ effect size are the key criteria for evaluating the structural model in PLS-SEM. Before conducting the analyses, the structural model must be examined for collinearity. The path coefficients might be biased if the estimation involves significant levels of collinearity among the predictor constructs. If the level of collinearity is extremely high (as indicated by a Variance Inflation Factor or VIF value of 5 or higher), one should consider removing one of the corresponding indicator(s). PERF5 “Realization of additional production capacity compared to the target” is deleted because the VIF value is higher than 5 (5.113).

**Figure 2:**
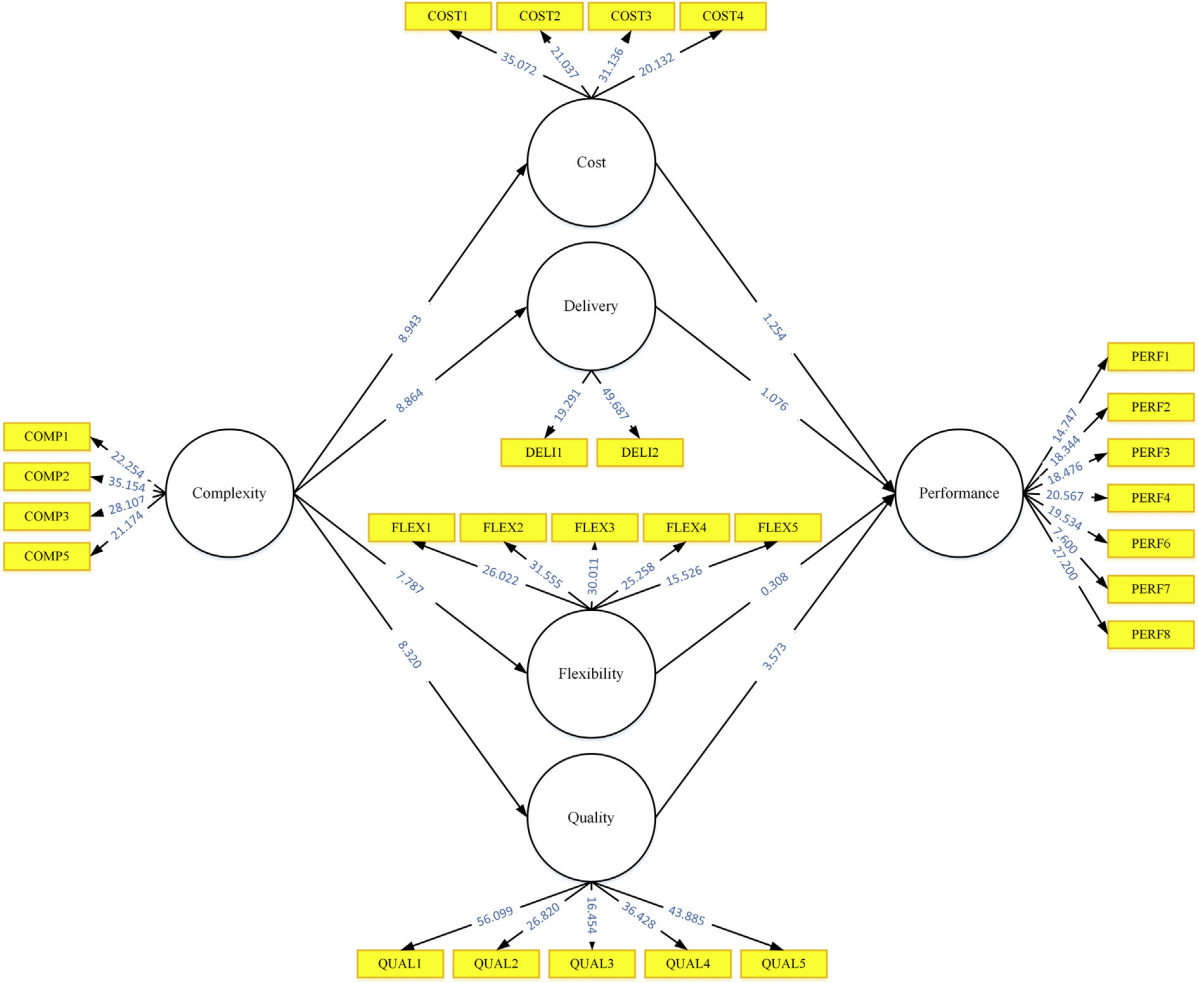
PLS-SEM results.

The path coefficients have standardized values between −1 and +1. The estimated path coefficients close to +1 represent strong positive relationships (and vice versa for negative values). All paths have a positive relationship with their dependent variable; however, not all variables are statistically significant (Table 5 and Figure 2). When the empirical t-value is larger than the critical value (the commonly used critical value is 1.96 for a significance level = 5%), the coefficient is significant at a certain error probability or significance level (Hair et al., 2016). As indicated by the results of PLS-SEM (Table 5, H1, H2, H3, H4, and H8 are accepted while H4, H5, and H6 are rejected). H1 “The higher the value of manufacturing complexity, the higher the value of cost strategy should be prioritized” is accepted because the t-statistics is higher than 1.96 and has a 0.685 positive path coefficient. H2 “The higher the value of manufacturing complexity, the higher the value of delivery strategy should be prioritized” is accepted because its t-value (8.864) is higher than the critical value and H2 has a positive coefficient (0.673). H3 “The higher the value of manufacturing complexity, the higher the value of flexibility strategy should be prioritized” is accepted because the path coefficient shows a positive relationship and the p-value is lower than 0.05. H4 “The higher the value of manufacturing complexity, the higher the value of quality strategy should be prioritized” is accepted because it has a positive path coefficient of 0.642 and t-statistics of 8.320. H8 “The manufacturing quality strategy has a positive impact on performance” is also accepted because the p-value is lower than 0.05. Meanwhile, H5, H6, and H7 are rejected because the t-statistics are lower than the critical value (1.96).

**Table 5:** Result summary of models.

	Path Coefficient	Standard Deviation	T Statistics	P Values	Decisions
H1 Complexity → Cost	0.685	0.080	8.943	0.000	Accepted
H2 Complexity → Delivery	0.673	0.076	8.864	0.000	Accepted
H3 Complexity → Flexibility	0.632	0.087	7.787	0.000	Accepted
H4 Complexity → Quality	0.642	0.081	8.320	0.000	Accepted
H5 Cost → Performance	0.133	0.107	1.254	0.215	Rejected
H6 Delivery → Performance	0.142	0.141	1.076	0.314	Rejected
H7 Flexibility → Performance	0.036	0.119	0.308	0.760	Rejected
H8 Quality → Performance	0.556	0.160	3.573	0.001	Accepted

The squared correlation between the actual and predicted values of a certain endogenous construct is used to compute R^2^, which is a measure of a model's predictive accuracy. The R^2^ value varied between 0 and 1, with greater values indicating more predictive accuracy. Endogenous latent variables with R^2^ values of 0.75, 0.50, or 0.25 can be classified as substantial, moderate, or weak (Hair et al., 2011 in Hair et al., 2016; Henseler et al., 2009). Table 6 shows the R^2^ values for all endogenous variables. The variables in the strategy manufacturing group (cost, delivery, flexibility, and quality) have R^2^ values between 0.40 and 0.50; thus, the predictive accuracy is considered moderate. The performance variable has an R^2^ value of 0.661, which indicates substantial predictive accuracy.

**Table 6:** R^2^ and Q^2^ results.

	R Square	Q Square
Cost	0.469	0.342
Delivery	0.453	0.337
Flexibility	0.400	0.278
Quality	0.412	0.329
Performance	0.661	0.430

The Q^2^ values that are greater than 0 indicate that the exogenous constructs have predictive relevance for the endogenous construct under consideration. The values of 0.02, 0.15, and 0.35, respectively, imply that an exogenous construct has a small, medium, or large predictive relevance for a given endogenous construct as a relative measure of predictive relevance (Q^2^). Table 6 shows that all variables have large predictive relevance because the scores are ~0.35.

### 4.2 Formed path

**Figure 3:**
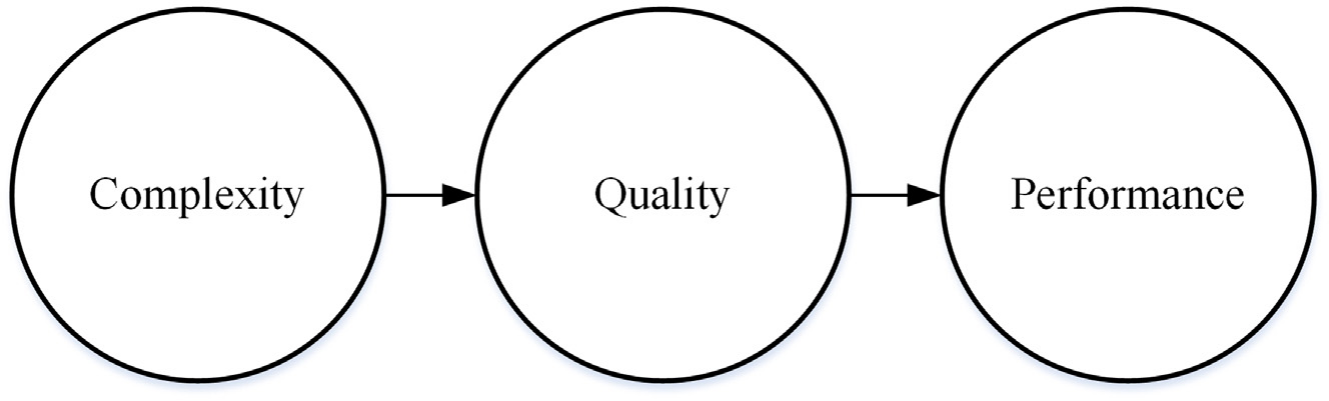
Formed path.

According to the significance test results in Table 5, five research hypotheses are accepted while three hypotheses are rejected. The formed path from the accepted hypotheses is shown in Figure 3.

Figure 3 indicates that the higher the manufacturing complexity, the quality strategy should be prioritized and the quality strategy has a positive impact on company performance.

## 5 Discussion

Analyzing and understanding manufacturing complexity allows us to develop and implement the correct strategies for the management of complexity (Efthymiou et al., 2016.). The acceptance of H1, H2, H3, and H4 in this study means that the higher the value of manufacturing complexity, the higher the value of manufacturing strategies (cost, delivery, flexibility, and quality) should be prioritized. This research supports the work of Ma et al. (2012) and Schuh et al. (2015) that the high-level complexity of manufacturing systems exerts a negative impact on the production process, production quality, reliability, throughput, and production time and disrupts system efficiency at the design, operation, maintenance, and management levels (Ma et al., 2012; Schuh et al., 2015). Therefore, the company needs to implement appropriate strategies to tackle these issues.

Complexity indicators used in this research were selected to fit the manufacturing industry. The statistically significant indicators based on PLS-SEM are (1)The sensitivity of the production method to product's changes compared to the initial conditions of demand, (2) Product changes have an impact on production methods, (3) Increased types and numbers of product components have an impact on the way of production, (4) There is a clear procedure in production. These indicators serve as guidelines for manufacturing companies in assessing their complexity. If these indicators have high complexity value, company have to adapt with prioritizing the strategies (cost, delivery, flexibility, and quality).

However, not all strategies have a significant positive impact on performance. Only the quality strategy of automotive component companies in Indonesia has a positive effect on performance (The acceptance of H8). The result supports those of previous studies that showed the positive effect of quality on performance (Amoako-Gyampah and Acquaah, 2008; Ferdows and De Meyer, 1990; Terjesen et al., 2010), particularly the positive impact of quality-based strategies on financial performance (Wood et al., 1990). The better the quality strategy is, the better the company performance will be. Therefore, we can conclude that the quality strategy for the automotive component companies in Indonesia is a top priority over the cost, delivery, and flexibility strategies. This result also supports Nurcahyo and Wibowo (2015), who stated that quality needs to be set as a top priority strategy. If a company's price is low and the quality of goods produced is also low, consumers will definitely go for companies with higher quality products (Nurcahyo and Wibowo, 2015).

Before applying quality improvement in the product, the management level needs to be improved by implementing Total Quality Management (TQM). The implementation of TQM is generally manifested in the form of implementing the Quality Management System (QMS) to increase customer satisfaction and improve company performance. Although several studies have proven that TQM improves the company's performance, the results vary due to many factors. The factors that influence the success of implementing QMS mainly can be seen from two factors, internal and external. Internal factors are about the company, for example, related to leadership, company capabilities, and human resources. These factors are within the company's control to improve and make continuous improvements. An example of the external factors is the QMS framework used by the company. This factor is beyond the company's control because the company only has to choose and use one of the available QMS frameworks. The most popular QMS framework in use around the world today is the ISO 9001 standard.

One of the efforts to improve the quality strategy is implementing ISO 9001 quality management system that has been proven by previous research to have a positive effect on the company performance. The ISO 9001 variables that significantly affect company performance in Indonesia are organizational leadership, customer requirement, defect prevention, continuous improvement, and supplier quality management (Nurcahyo et al., 2021). In doing so, managers need to ensure that no dualism system exists between the company's daily systems and the ISO 9001 quality system. Managers need to run an ISO 9001 quality management system as part of the daily routine activities of the company and not only during auditing by the certification body. This action is needed to reap the benefits of ISO 9001, especially in relation to improving product quality and company performance in general. Moreover, managers need to be concerned about the company's internal capabilities, especially the dynamic capabilities of manufacturing, because these capabilities affect the product quality strategy. These internal factors have a more positive influence on quality strategy selection than external factors. The development of human resources related to skills, knowledge, and technical experience also requires consideration. The lack of human resource capabilities related to technical aspects could affect product quality.

Suppliers' performance also plays a role in product quality. When a new product is reintroduced, a product is returned, or a product is modified, manufacturers must deal with changing consumer demands. Quick delivery and high-quality raw materials from suppliers, as well as their participation in quality assurance programs, are critical to accomplishing this. As a result, involving suppliers enhances efficiency and increases the manufacturer's ability to deal with changing market conditions. The researcher recognized that quality management (QM) should be extended to both upstream and downstream supply chain partners, rather than being firm-centric to cope with a high-velocity business environment (Phan et al., 2019). Supplier involvement is a form of upstream quality control that helps companies ensure the quality of their raw materials and make use of their suppliers' quality assurance capabilities (Phan et al., 2019). It is also emphasized that delivery by suppliers contributes significantly to competitive performance, especially volume flexibility and product mix flexibility. This necessitates continuous contact between the manufacturer and its suppliers. Supplier involvement in TQM practices accelerates supplier collaboration and creates a commitment to supply high-quality products from the supplier. As a result, supplier involvement in quality tends to lower the rate of input rejection.

Quality indicators illustrate suitability and consistency so that it is defined as consistency in producing products with a low defect level and conformance to requirements. Defects typically occur during the manufacturing process when production is disrupted (Phan et al., 2019). In various manufacturing processes, quality control and improvement at the process level are critical for achieving defect-free products (Bataineh and Al-Dwairi, 2011). TQM practices decrease defects and avoid rework to reduce a redundant step in the production process. Therefore, the cycle time is shortened, and manufacturers can respond faster to consumer demands with greater flexibility and capability (Phan et al., 2019). Migrating from a traditional factory to a smart factory also beneficial for reducing the defect rate, such as the research by Lee et al. (2021). Their fault detection technologies have a 96.9% accuracy rate and are expected to be a promising smart-factory tool for lowering defect rates and production costs. Another way to reduce the defect rate is by using the quality tools such as seven tools and SPC.

In quality control, statistical methods such as seven tools and SPC are widely utilized. Seven tools are quality control tools that include check sheet, histogram, Pareto chart, Cause and effect chart, graphical tools, scatter plot, and control chart. The use of seven tools in Indonesian automotive manufacturers is not something new. Putri and Yusof (2011) show that Indonesian manufacturers have a high implementation level for almost all the seven basic tools, although there were some obstacles such as lack of understanding of basic knowledge and lack of commitment from top management. In addition, the implementation of SPC tools, mainly control charts and capability assessment, can significantly facilitate quality improvement by reducing process variability and shifts in the process mean (Bataineh and Al-Dwairi, 2011). A control chart, which graphically represents process data and indicates whether the process is under statistical control or not, is traditionally the most powerful SPC tool. SPC's main goal is to give signal when a process changes, such as when the mean deviates from the target value or when the variability increases (Škulj et al., 2013). When a signal shows that the process is changing, the machine operator must take corrective action. If the set quality requirements are not satisfied, for example, a batch of products will be rejected. A maximum number of defective products in the sample is typically used as an acceptance criteria. If the quantity of defective products in the sample exceeds the acceptance criteria, the sample must be rejected, and the entire batch must be inspected 100 percent. The discovered defect products must be examined in order to determine the root causes of the problems (Oppermann et al., 2001). As a result of SPC tools implementation, an expected reduction in the number of defective products by 29% relative to the stage before implementation was achieved (Bataineh and Al-Dwairi, 2011).

Lastly, apart from quality suppliers, quality raw materials, and controlled processes, the machines used in production also need attention (Gu et al., 2017). Maintenance, production, and quality are strongly linked to each other. Predictive maintenance is an effective way to eliminate the potential failures, ensure the manufacturing system's stable operation, and further improve the reliability of the manufacturing system and the quality of manufactured Products (Gu et al., 2017). The most recent and comprehensive concept in maintenance is Total Productive Maintenance (TPM). TPM is a system for maintaining and improving the quality of production through the maintenance of equipment such as machinery and work tools. The implementation of TPM is expected to ensure all equipment and production machines are operating in the best condition to avoid any damage that causes defective products or delays in the production process.

By implementing these quality strategies, it is expected that the company will get an improvement in performance such as product quality, number of sales, market share growth, lower production costs, cost reduction for raw materials, reliability in delivering products on time, and the number of product variations that the company can make.

## 6 Conclusions

The results of this study will aid automotive component companies in developing their manufacturing strategies. The data collected from 72 automotive component companies in Indonesia and processed via PLS-SEM indicate that the higher the value of manufacturing complexity, the higher the value of strategies should be prioritized, such as those related to cost, delivery, flexibility, and quality. However, only the quality strategy exerts a statistically significant effect on the performance of automotive component industries in Indonesia. The formed path between complexity, strategy, and performance shows that the higher the value of manufacturing complexity, the quality strategy needs to be prioritized by managers of automotive component companies in Indonesia to improve the performance.

The manufacturing companies need to establish the correct strategies in addressing the increase of manufacturing complexity. Related to the delivery strategy, companies are recommended to implement the QRM and reduce the lead time so the products could be delivered faster than competitors. Related to the flexibility strategy, companies are suggested to combine TQM and JIT production practices, especially for process control and setup time reduction, supplier involvement and JIT delivery by suppliers, customer involvement and JIT link with customers. Related to cost strategy, companies are advised to reduce the production and distribution costs, inventory, and increase the machine utilization and capacity (reduce dependence on humans). Furthermore, the quality strategy should be the priority of manufacturing companies. Companies are suggested to reduce the defect rate, improve product quality, improve suppliers' quality and implement ISO 9001 quality management system.

The novelty of this study lies in its detailed examination of the relationship between manufacturing complexity, manufacturing strategies, and manufacturing performance, particularly in Indonesia. Such investigation helps fill the existing research gaps. One of the limitations of this study is that it only covers one industry: the automotive component industry. The similarity between the characteristics of the electronics industry and the automotive industry lies in the dominant role of component suppliers. Therefore, future related research should explore the electronics industry.

## Declarations

### Author contribution statement

Hendri Dwi Saptioratri Budiono: Conceived and designed the experiments; Analyzed and interpreted the data; Wrote the paper.

Rahmat Nurcahyo: Conceived and designed the experiments; Analyzed and interpreted the data; Contributed reagents, materials, analysis tools or data; Wrote the paper.

Muhammad Habiburrahman: Performed the experiments; Analyzed and interpreted the data; Contributed reagents, materials, analysis tools or data; Wrote the paper.

### Funding statement

This work was supported by Hibah PPI Q2 Universitas Indonesia 2021.

### Data availability statement

Data will be made available on request.

### Declaration of interests statement

The authors declare no conflict of interest.

### Additional information

No additional information is available for this paper.

## Appendix A Supplementary data

Appendix - Questionnaire.docx Appendix - Questionnaire.docx
