# Supplementary material for: Relationship between manufacturing complexity, strategy, and performance of manufacturing industries in Indonesia
Source: Heliyon. 2021 Jun 5;7(6):e07225. doi: 10.1016/j.heliyon.2021.e07225 (PMC8214087; doi:10.1016/j.heliyon.2021.e07225)
Supplement: Appendix - Questionnaire.docx [file mmc1.docx]

**RELATIONSHIP BETWEEN MANUFACTURING COMPLEXITY, STRATEGY, AND PERFORMANCE OF MANUFACTURING INDUSTRIES IN INDONESIA**

**Hendri Dwi Saptioratri Budiono, Rahmat Nurcahyo, and Muhammad Habiburrahman**

**Appendix 1 -** Questionnaire.

Company Data (data will not be displayed at the writing of the article)

Components / products made by the Company (can be filled in more than 1): ........................

......................................................................................................................................

Company products for purposes (can be filled in more than 1):

|  | Japanese automotive |  |  | Europe automotive |  |  | Korean automotive |
| --- | --- | --- | --- | --- | --- | --- | --- |

|  | American automotive |  |  | Others (mixed) ...................... |  |  |  |
| --- | --- | --- | --- | --- | --- | --- | --- |

Company age:

|  | < 5 years |  |  | 5-10 years |  |  | > 10 years |
| --- | --- | --- | --- | --- | --- | --- | --- |

Number of employees:

|  | < 100 |  |  | 100-500 |  |  | > 500 |
| --- | --- | --- | --- | --- | --- | --- | --- |

Respondent Data (data will not be displayed in writing articles)

Age:

|  | <30 years old |  |  | 30-40 years old |  |  | > 40 years old |
| --- | --- | --- | --- | --- | --- | --- | --- |

Position:

|  | Director |  |  | Manager |  |  | Asisten Mgr |
| --- | --- | --- | --- | --- | --- | --- | --- |

|  | Supervisor |  |  | Other: please specify................................. |
| --- | --- | --- | --- | --- |

Work experience in the automotive industry (both at this company and other automotive companies):

|  | <5 years |  |  | 5-10 years |  |  | > 10 years |
| --- | --- | --- | --- | --- | --- | --- | --- |

Division / Department currently working:

|  | Manufactur |  |  | Production |  |  | Engineering |
| --- | --- | --- | --- | --- | --- | --- | --- |

|  | Marketing |  |  | Other: please specify................................. |
| --- | --- | --- | --- | --- |

Other data

Company name : ......................................................................................................................

Company's address : ......................................................................................................................

1. The following questions are related to the **Complexity of Manufacturing Systems**

Complexity has been identified as a property of a manufacturing system which is divided into 2, namely static and dynamic. Static (or structural) complexity represents a time characteristic independent of the manufacturing system and focuses on the type of sub-system and the strength of the interconnections. Static complexity is divided into three variables: System structure function, variation, interconnection. Dynamic (or operational) complexity is an operational characteristic of a system and involves both timing and randomness aspects. Dynamic complexity is divided into three, namely; idle, busy and failed.

Please answer all the following questions. There are no right or wrong answers.

|  | **Totally**  **Disagree** | | **Agree** | |  | **Totally**  **Agree** | |
| --- | --- | --- | --- | --- | --- | --- | --- |
| *Which of the following factors happened in the company* |  |  |  |  |  |  |  |
| 1. The sensitivity of the production method to changes in the product compared to the initial conditions of demand (COMP1) | 1 | 2 | 3 | 4 | 5 | 6 |  |
| 1. Product changes have an impact on production methods (COMP2) | 1 | 2 | 3 | 4 | 5 | 6 |  |
| 1. Increased types and numbers of product components have an impact on the way of production (COMP3) | 1 | 2 | 3 | 4 | 5 | 6 |  |
| 1. Uncertainty in increasing product variety (COMP4) | 1 | 2 | 3 | 4 | 5 | 6 |  |
| 1. There is a clear procedure in production (COMP5) | 1 | 2 | 3 | 4 | 5 | 6 |  |
| 1. There are deviations in the use of resources scheduled with realization (COMP6) | 1 | 2 | 3 | 4 | 5 | 6 |  |
| 1. Difficulty in determining key performance indicators (KPI) (COMP7) | 1 | 2 | 3 | 4 | 5 | 6 |  |

1. The following questions relate to **Manufacturing Strategy**

Manufacturing strategy is a company's priority in competing, whether to give priority to cost factors, delivery factors, flexibility factors or quality factors. Priority can be more than one.

Please answer all the following questions. There are no right or wrong answers.

|  | **Not a priority at all** | | **Priority** | |  | **Top Priority** | |
| --- | --- | --- | --- | --- | --- | --- | --- |
| *Which of the following factors is the main priority and which is not a priority for the company* |  |  |  |  |  |  |  |
| ***Cost*** |  | | | | | |  |
| - Reduction of production costs (COST1) | 1 | 2 | 3 | 4 | 5 | 6 |  |
| - Reduction in material / raw material costs (COST 2) | 1 | 2 | 3 | 4 | 5 | 6 |  |
| - Reduction of overhead costs (COST 3) | 1 | 2 | 3 | 4 | 5 | 6 |  |
| - Reduction of inventory / stock (COST 4) | 1 | 2 | 3 | 4 | 5 | 6 |  |
| ***Delivery*** |  | | | | | |  |
| - Faster product delivery (DELI1) | 1 | 2 | 3 | 4 | 5 | 6 |  |
| - Delivery of products on time (DELI 2) | 1 | 2 | 3 | 4 | 5 | 6 |  |
| ***Flexibility*** |  | | | | | |  |
| - Reduction of production lead time (FLEX1) | 1 | 2 | 3 | 4 | 5 | 6 |  |
| - Reduced machine set up time (FLEX2) | 1 | 2 | 3 | 4 | 5 | 6 |  |
| - Production flexibility in relation to the variable production schedule (FLEX3) | 1 | 2 | 3 | 4 | 5 | 6 |  |
| - Production flexibility in terms of fluctuating production volume levels (FLEX4) | 1 | 2 | 3 | 4 | 5 | 6 |  |
| - Machine flexibility regarding variable product variations (FLEX5) | 1 | 2 | 3 | 4 | 5 | 6 |  |
| ***Quality*** |  | | | | | |  |
| - Reduction of the defect rate (QUAL1) | 1 | 2 | 3 | 4 | 5 | 6 |  |
| - ISO 9001 certification (QUAL2) | 1 | 2 | 3 | 4 | 5 | 6 |  |
| - Improved supplier quality (QUAL3) | 1 | 2 | 3 | 4 | 5 | 6 |  |
| - Use of quality tools for example: 7 tools, SPC (QUAL4) | 1 | 2 | 3 | 4 | 5 | 6 |  |
| - Improved product quality (QUAL5) | 1 | 2 | 3 | 4 | 5 | 6 |  |

1. The following questions are related to **Company Performance**.

Please answer all the following questions.

|  | **Much smaller than the target** | |  |  |  | **Much bigger than the target** | |
| --- | --- | --- | --- | --- | --- | --- | --- |
| *Provide an assessment of the* ***company's performance*** *when compared between realization and targets for* ***the last 2 years****.* |  |  |  |  |  |  |  |
| - Realization of increasing number of sales compared to target (PERF1) | 1 | 2 | 3 | 4 | 5 | 6 |  |
| - Realization of market share growth compared to target (PERF2) | 1 | 2 | 3 | 4 | 5 | 6 |  |
| - Realization of production costs when compared to the target (PERF3) | 1 | 2 | 3 | 4 | 5 | 6 |  |
| - Realized cost reduction for raw materials compared to target (PERF4) | 1 | 2 | 3 | 4 | 5 | 6 |  |
| - Realization of additional production capacity compared to the target (PERF5) | 1 | 2 | 3 | 4 | 5 | 6 |  |
| - Realization of reliability in delivering products on time, when compared to the target (PERF6) | 1 | 2 | 3 | 4 | 5 | 6 |  |
| - Realization of product quality, when compared to the target (PERF7) | 1 | 2 | 3 | 4 | 5 | 6 |  |
| - Realization of the number of product variations that the company can make, when compared to the target (PERF8) | 1 | 2 | 3 | 4 | 5 | 6 |  |
